# Supplementary material for: Are carbon emissions trading and green financial instruments synergistic? -Comprehensive quantitative research based on content analysis
Source: PLoS One. 2024 Mar 7;19(3):e0298601. doi: 10.1371/journal.pone.0298601 (PMC10919601; doi:10.1371/journal.pone.0298601)
Supplement: S1 Data — (ZIP) [file pone.0298601.s001.zip › Data/地级市Z_F工作报告.zip!/║╙─╧/╚2├┼╧┐╩╨/└┤╫╘╣1⁄2╓┌║┼í╛┬φ┐╦╩2╛▌═°í┐_2005.docx]

各位代表：

　　现在，我代表市人民政府向大会作政府工作报告，请予审议，并请各位政协委员和其他列席人士提出意见。

一、2004年工作回顾

　　刚刚过去的一年，市政府在省委、省政府和市委的正确领导下，在市人大、市政协的监督支持下，紧紧围绕“加快发展，维护稳定，狠抓落实”的工作大局，团结和带领全市人民，求真务实，开拓创新，圆满完成了市四届人大五次会议确定的各项任务，经济和社会发展的11项主要预期目标全面实现。据初步测算，全市生产总值达到273亿元，按可比价格计算，比上年增长14%。

　　大力实施项目带动战略，经济发展势头强劲。预计全社会固定资产投资完成111亿元，比上年增长46%。项目建设呈现出前所未有的喜人局面，全年投资3000万元以上的工业项目58个，以“三大项目”为龙头、24个投资超亿元的骨干支撑项目进展顺利。义马气化厂扩建、渑池韶星氢氧化铝等36个项目开工建设，义马锦江电厂、惠能热电、天元再生铝、灵宝鑫源果蔬加工等29个项目建成投产。

　　加快工业结构调整步伐，产业基地建设初具规模。煤炭、铝土矿资源整合稳步实施，原煤产量、发电量均创历史新高，煤化工、铝工业产业链逐步拉长，黄金精深加工取得新进展，4家企业进入省百家重点扶持企业行列。预计规模以上工业完成增加值97.6亿元，比上年增长20%。经济效益综合指数151.79%，提高4.13个百分点；实现利税33.9亿元，增长16%。

　　突出特色城市建设，城镇体系逐步完善。中心城市总体规划和城镇体系规划编制完成，“一城三区”城市框架初显形象。快速通道二期、国道209线市区段改造和4条城市道路改建通车，上阳大桥和崤山路、黄河路两个立交桥基本完工，青龙大坝完成后续工程，苍龙大坝具备蓄水条件，污水处理厂一期投入试运行。大力进行城市环境综合整治，创建园林城市活动深入开展，南山北岭、三河两岸绿化工作全面铺开，顺利通过省级卫生城市复检，城镇带建设步伐加快。

　　坚持用工业理念发展特色农业，产业化水平不断提高。牧、果、林、烟等支柱产业进一步壮大，优质苹果等特色农产品打入国际市场，果品、林板和畜牧等综合加工形成规模。建成标准化生产基地100万亩，退耕还林达到118.9万亩。市农产品质量检测中心全省首家通过“双认证”，农业信息化在全国领先。

　　努力完善服务功能，第三产业快速增长。改造提升综合服务设施，连锁店和超市经营迅速扩张。预计全社会消费品零售总额比上年增长13.7%。第十届国际黄河旅游节取得圆满成功，实施重大旅游合作项目3个，接待国内、入境游客分别增长17.1%和10.1%。

　　充分发挥财政金融职能，经济金融协调发展。地方财政一般预算收入完成11.68亿元，比上年增长28.6%，净增近3亿元，创历史最高水平。金融机构各项存款余额比年初增加29亿元，贷款余额增加23.9亿元，增幅和绝对额均居全省前列。

　　积极推进改革开放，发展活力明显增强。贯彻实施《行政许可法》，坚持改革行政审批制度，取消行政审批事项71项，政府机构改革顺利完成，行政效能不断提高。国有企业改革完成20家，分离企业办社会职能实现平稳过渡，天元铝业、湖滨果汁境外成功上市。粮食流通体制和农村信用社改革取得实效，乡村财务管理体制改革、乡财县管逐步到位。经济外向度明显提高，吸收外商直接投资5758万美元，比上年增长37.1%；出口创汇1.12亿美元，增长18%；劳务输出16万人。非公有制经济快速发展，预计上缴入库税金增长17.5%。县域经济不断壮大，预计生产总值增长14%。

　　统筹发展各项社会事业，人民生活质量大为改善。纤维素酶、生物肥料等多项高新科技项目全国领先，科技对经济增长的贡献率达48%。继续加大教育投入，新改扩建校舍18万平方米，职业教育、成人教育协调发展。疾病控制、公共卫生项目建设和农村新型合作医疗试点进展顺利，城乡医疗卫生条件得到改善。人口和计划生育工作不断加强，人口自然增长率4.05‰。人才、就业和社会保障工作取得新成绩，城镇登记失业率3.4%，做到了“两个确保”和对城镇“低保”对象的应保尽保。稳定解决了2.5万贫困人口的温饱问题和5.1万人的饮水困难，灾区倒房重建8500余间，实现了“四有一不”的救灾目标。省道247线改建完工，新改建县乡公路400公里，新增通油路行政村60个。预计农民人均纯收入2496元，比上年增长11.1%；城镇居民人均可支配收入7234元，增长8%。审计、物价、外事、侨务、对台、民族、宗教、农机、气象、人防、档案、史志、无线电、河务移民、防震减灾和残疾人事业等有了新进步。

　　切实加强社会主义精神文明和民主法制建设，构建和谐社会有序推进。认真贯彻党的十六届三、四中全会精神，深入开展“三好一强”活动，“双创”和“两抓一促”取得实效，广播电视和新闻出版取得新成绩，文化、体育活动蓬勃开展，“双拥”和民兵预备役工作得到加强。严格执行市人大及其常委会通过的决定、决议，办理人大代表建议96件、政协委员提案295件。坚持依法治市，以创建综合治理先进市为载体，加强治安防控体系建设，打击刑事和经济犯罪活动取得新成果。认真落实党风廉政建设责任制，反腐纠风成效明显。开展安全生产专项整治和督查，安全形势趋于平稳。健全工作机制，集中处理信访积案，保持了社会大局稳定。

　　各位代表，过去的一年，是谋发展、保稳定、抓落实取得显著成效的一年，是工业化、城镇化和农业现代化实现快速发展的一年，更是为跻身中原崛起“第一梯队”、全面建设小康社会打下坚实基础的一年。这些成绩的取得，是省委、省政府和市委正确领导的结果，是市人大、市政协大力支持的结果，是全市人民奋力拼搏的结果。值此，我代表市人民政府，向工作在全市各条战线上的广大工人、农民、知识分子、干部、人民解放军指战员、武警官兵以及社会各界人士致以崇高的敬意和衷心的感谢！

　　在肯定成绩的同时，我们也清醒地看到，在经济社会发展中还存在不少困难和问题：一是经济结构还不尽合理，电价升高、资金短缺等制约因素没有得到有效缓解，生产成本上升，工业亏损增加。二是重点项目建设进展不平衡，个别项目推进较慢。三是环境质量不够理想，提升城市整体形象的任务仍很艰巨。四是经济发展环境需要进一步优化，个别机关工作人员服务意识不强，仍存在设卡、刁难和变相收费等不良现象。同时，还有一些地方治安形势不好，整顿和规范市场经济秩序以及社会稳定工作有待继续加强。对这些问题，我们一定高度重视，在今后的工作中切实加以解决。

二、2005年政府工作的总体要求和主要任务

　　今年政府工作的总体要求是：以邓小平理论和“三个代表”重要思想为指导，认真贯彻党的十六大和十六届四中全会精神，用科学发展观统领经济社会发展全局，紧紧围绕跻身中原崛起“第一梯队”和提前实现全面建设小康社会的总体目标，突出加快经济发展与构建和谐社会两大任务，牢牢把握“加快发展，维护稳定，狠抓落实”的工作大局，大力实施“扩大开放，依托提升，项目带动”发展战略，继续坚持“发挥优势，培育特色，优化结构，持续发展”的经济工作方针，服从大局，审时度势，突出重点，顺势而为，努力转变经济增长方式，全面提高国民经济的整体素质和竞争力。进一步加强社会主义精神文明和民主法制建设，努力实现经济社会全面协调可持续发展。

　　按照上述要求，今年经济和社会发展的主要预期目标是：全市生产总值增长11%；全社会固定资产投资增长22%；社会消费品零售总额增长12%；吸收外商直接投资增长13%，出口创汇增长10.5%；地方财政一般预算收入增长13%；新增就业岗位3.2万个；农民人均纯收入增长5%以上，城镇居民人均可支配收入增长8%以上；人口自然增长率控制在6‰以内。

　　 为完成以上目标，要着力抓好八个方面的工作：

　　（一）强力推进项目建设，努力完善基础设施

　　集中抓好“421”工业重点项目。全年计划安排投资在3000万元以上的重大项目40个以上。要采取得力措施，科学安排施工，加快“三大项目”建设进度，争取开曼铝业和东方希望两大氧化铝项目一期年内建成，大唐电厂明年上半年投入运行。抓好义马开祥甲醇等一批续建项目，确保义马综合利用电厂、中迈电厂、渑池液晶显示屏、三门峡天意沸石等20个以上项目建成投产，促使三门峡戴卡轮毂、天元再生铝二期、湖滨果汁饮料二期及乳制品加工等10个以上项目开工建设。同时，要加快推进项目前期工作，务求义马电厂4×60万千瓦机组一期等重大项目取得突破；重点储备一批对经济发展有重大带动作用的大中型项目，为经济发展备足后劲。

　　突出抓好“四路三库”重大基础设施项目。大力支持郑（州）西（安）快速客运专线三门峡段的建设，完成省道314、318、322线改建路基和桥涵工程。积极争取亚行贷款，西段村水库完成大坝主体2/3以上；多方筹措资金，加快卫家磨水库建设进度；按照市场运作模式，尽快完成沟水坡水库除险加固，并利用窄口水源建设两库贯通的供水工程，为三门峡西区建设提供水源保证。

　　积极争取国债项目和资金。紧紧抓住国家加强薄弱环节建设的有利时机，努力将农业、卫生、教育、基础设施、生态环境等项目列入国家和省国债计划。加强对国债项目建设的监督和管理，确保市疾病控制中心大楼建设、市第二人民医院改建完成投入使用。实施灵宝、湖滨、陕县饮水安全项目和以坝系建设为主的水土保持项目，发展节水灌溉4500亩，农业综合开发治理2万亩。完成县乡公路改造100公里，新增通油路行政村30个。加大金融对项目建设的支持力度，完善中小企业担保体系，争取上级扶持资金，为经济建设提供资金保障。

　　 （二）重点实施工业结构的战略性调整，进一步转变经济增长方式

　　大力发展循环经济，加快产业聚集。坚持“减量化、再利用、再循环”的原则，统筹规划产业发展，搞好资源整合和优化配置，围绕能源、煤化工、铝工业、黄金生产加工和林果业生产加工五大基地建设，调整产业结构，拉长产业链条，形成规模优势，尽快建成以310国道为轴线的东西“工业走廊”，走出一条经济、社会和环境相协调的新型工业化之路。

　　加快发展高附加值的加工制造业，抓好产业优化升级。积极引进国内外先进技术和人才，以制造业信息化为突破口，改造提升精密量仪、成套设备、工程机械等传统产业，发展以铝、黄金深加工为主的现代加工制造业，形成先进制备基地。严格执行国家质量、技术、环保等准入标准，大力开发新产品，抓好新材料、生物工程、现代化工、医药等项目的实施，推动高新技术产业发展。

　　努力培育和壮大重点企业，增强工业经济的核心竞争力。抓好义煤、天元、金渠、湖滨果汁等重点企业的发展，壮大企业规模，充分发挥带动作用；选择一批符合国家产业政策、符合区域经济规划的骨干企业，指导筹划项目，争取政策支持。进一步把企业扶大做强，力争今年销售收入超20亿元的企业达到2个，超10亿元的达到5个以上。

　　切实加强监测和指导，建立工业运行协调机制。密切关注工业经济发展态势，努力克服不利因素影响，有效缓解煤电油运紧张局面，帮助企业扭亏增盈。强化企业管理，深化内部改革，建立技术创新和激励约束机制。大力引进优秀人才，重视对企业家以及高级经管人员的培养，提高经营管理水平。

　　 （三）积极实施中心城市带动战略，加快县域经济发展

　　进一步提升城市品位。突出产业定位和文化、生态特色，全面实施城市总体规划。重点搞好涧河四期治理、涧河带状公园和苍龙大坝建设，集中进行快速通道两侧环境整治和绿化美化，打造贯穿“一城三区”的景观走廊，展示自然山水城市的整体形象。扎实推进城市集中供热、供气两大工程，力争年底投入运行。加快城市供水、污水管网和垃圾处理工程进度，完善综合服务设施，增强中心城市的辐射带动力。

　　打好创建园林城市攻坚战。编制和实施绿地系统规划。整修城市道路，加快主干道和过市公路、铁路的绿化达标步伐。推进园林绿化，丰富青龙湖区、虢国公园、中日友好园的园林内涵。全面铺开中部生态区、南山北岭、北部沿黄等大环境绿化，努力营造城市森林。深入开展锅炉除尘脱硫、火电厂脱硫、机动车尾气治理、工业废水达标排放等6大专项治理，使市区环境质量有明显改善。强化城市管理，积极探索和完善相对集中行政处罚权工作，提高综合管理水平，争取年底通过省级园林城市验收。

　　加快城镇带建设步伐。抓紧制定310国道沿路城镇带实施方案，研究配套政策，继续抓好义马、渑池城市建设对接，加大灵宝北区、卢氏新区开发力度，推进副中心城市建设；重点镇建设要结合产业布局调整，发展特色产业，突出一镇一业，把沿路城镇带建成经济隆起带。

　　发展壮大县域经济。用足用好上级扶持政策和资金，重点支持环保、旅游基础设施和特色工业项目建设，突出抓好义马的能源、煤化工，渑池的煤电铝一体化、建材、食品，市区的林果、机械、医药和铝的精深加工，陕县的热电、化工、农副产品加工，灵宝的黄金、化工、林果生产加工，卢氏的绿色食品、矿业、旅游等，做大主导产业，增强县域经济实力。

　　 （四）高度重视和加强“三农”工作，促进农业和农村经济快速发展

　　加大对“三农”的支持力度。坚持把解决好“三农”问题作为重中之重，不折不扣地落实党在农村的各项政策，全面取消农业税，继续实行对种粮农户直接补贴，有效增加农民收入，保护和调动广大农民的生产积极性。加大财政投入，完善农业服务体系，强化新技术的引进、推广和应用，扶持农机大户，提高农业综合生产能力。深入开展农村收费专项治理，切实减轻农民负担。

　　抓好特色产业基地建设。围绕发展牧、果、林、烟等支柱产业，建设50万头瘦肉型猪、25万头优质肉牛、25万只优质肉羊、50万只卢氏绿壳蛋鸡等畜牧生产加工基地；新发展无公害苹果基地10万亩，实施人工造林16万亩，封山育林15万亩。重点抓好25万亩优质烟叶、10万亩无公害蔬菜、4200万袋食用菌以及万亩玫瑰药材基地建设，加快形成区域性主导产业。

　　大力推进农业产业化、标准化、信息化。围绕发展特色农业，壮大湖滨果汁、缘份果业、灵宝果品、河洛板业、义华养殖、天顺肉牛、金红烟草等龙头企业，拉长果牧林烟产业链。完善市、县农产品检测服务网络，搞好原产地域产品保护认证和绿色食品生产，打造SOD苹果、“三门峡”牌烟叶等特色名牌产品。推动农业信息化“进村、入户、到企”，突出抓好10个示范村、30个龙头企业等信息网点。规划建设一批农产品专业批发和集贸市场，发展新型农村专业合作经济组织和农民经纪人队伍，开拓国内外市场。

　　 （五）强化城市产业支撑，提升第三产业水平

　　加快发展现代服务业。按照适度超前、发展大服务的方向，完善政策措施，构建高起点、多层次的现代服务业体系。进一步优化环境，加快发展金融、保险、信息、咨询、法律等服务业。加强物业管理、社区服务等配套建设，规范发展城市新兴服务业。

　　大力发展现代物流业。充分发挥区位和交通优势，改造提升专业批发市场，拓展公路、铁路货运市场，壮大物流运输和配送规模，建设东接郑州、西连西安、北通山西的现代化物流园区。依托现有流通商贸企业，改制重组2—3个大型商贸企业集团。大力发展以连锁经营为主的现代流通业，吸引国内外知名商贸企业集团来我市发展，增强在黄河金三角地区的市场辐射力。

　　继续发展旅游业。坚持把旅游业作为一项新兴支柱产业来培育，整合旅游资源，按照市场运作，全方位进行旅游产品的组合包装和形象设计，加大宣传推介促销力度，打响三门峡“文化山水”旅游品牌。重点提升虢国博物馆、函谷关景区品位，加快空相寺建设步伐。按照“谁投资、谁受益”的原则，融资实施仰韶、豫西两个大峡谷和亚武山景区等合作项目。筹备好与日本北上市缔结友好城市二十周年纪念活动，全面提升第十一届国际黄河旅游节的整体水平，进一步扩大三门峡的知名度和对外影响力。

　　（六）坚持改革开放，增强发展活力

　　进一步深化改革。国有企业改革要继续落实责任，加快进度，确保改革规范、有序进行。完善国有资产监管体制，强化经营收益管理，实现保值增值。流通商贸企业改革要积极运用国企改革的有关政策和经验，深入调研，制定方案，一企一策，年内大部分完成。投资体制改革要彻底改革项目审批制度，规范核准制和备案制，落实企业投资自主权，充分调动全社会投资的积极性。进一步抓好市政公用行业、交通、水利等建管体制和科技、教育、文化、卫生等社会事业领域的改革，抓紧落实方案，积极稳步推进。

　　全面扩大对外开放。改善投资环境，健全激励机制，扩大招商引资的领域和范围。引导机关事业单位干部、企业家、中介机构人员通过各种对外交往方式，开展全民招商引资活动，提高洽谈项目成功率。积极帮助更多的企业取得外贸经营权，继续巩固铝及铝制品、果汁、刚玉砂、纺织品等拳头产品出口，支持高新技术和机电产品、特色农产品打入国际市场。加强区域经济技术合作，鼓励企业到市外谋求发展，有效承接东部地区产业转移，开拓西部发展空间。

　　放手发展非公有制经济。完善和落实支持非公有制经济发展的政策措施，进一步放宽非公有资本进入基础设施、垄断行业以及法律、法规没有禁止的其他行业和领域，鼓励非公有资本参与国有、集体企业改制和重组。选择符合产业政策、市场前景好的中小企业，纳入产业规划，予以重点扶持，发挥示范带动作用，激发经济发展活力。

　　（七）坚持以人为本，努力构建和谐社会

　　积极做好就业和社会保障工作。认真落实各项就业优惠政策，抓好劳动力市场“三化”建设，多渠道开展就业培训，促进失业人员自主创业和自谋职业。重视开发公益性岗位，安排“4050”人员就业。扎实做好大中专毕业生就业、复员转业军人安置、城镇新增劳动力和农村富余劳动力就业工作，发展劳务经济。集中筹措国有企业下岗人员基本生活保障资金，保证基本生活费和“三金”缴纳，稳妥推进与失业保险并轨，确保企业离退休人员养老金按时足额发放。依法协调劳动关系，大力开展社会保险扩面征缴，使征缴率稳定在90%以上。抓好动态管理和分类施保，做到城市“低保”对象应保尽保。全面开展农村特困户救助，加快建立农村最低生活保障制度。

　　切实加强公共财政体系建设。围绕壮大市级经济，高起点、大力度新上财源项目。依法强化税收征管，确保财政收入稳定增长。加强非税收入收缴管理，增加政府可用财力。坚持“一是吃饭、二是还账、三是建设”的原则，调整优化支出结构，保重点项目，保重点支出，集中财力办大事。进一步完善部门预算和财政国库集中收付制度，严格收支两条线管理，扩大政府采购范围和规模。继续巩固乡财县管和乡村财务管理制度改革成果，推进依法理财。建立政府债务预警控制制度，防范和化解财政风险。

　　认真解决关系人民群众切身利益的问题。扎实推进科技扶贫和搬迁扶贫，完成400户搬迁，解决和巩固2.3万贫困人口温饱问题，解决农村安全饮水3万人。加强黄河库区塌岸治理，妥善安置好水库移民。强化自然灾害监测预报和减灾、救灾体系建设，增强抗灾能力。加大清理拖欠工程款和农民工工资力度，确保完成清欠目标。进一步规范土地征用、城镇拆迁、企业改制等工作，切实保护城乡居民的合法权益。完善并落实国家资助经济困难学生的各项政策。继续整顿和规范市场经济秩序，严厉打击制售假冒伪劣食品、药品、农资等违法犯罪活动，保护人民群众的切身利益。

　　加快推进社会事业发展。健全和完善公共卫生体系，加强非典、禽流感等传染病的监测和防治，巩固卢氏新型农村合作医疗试点成果，提高医疗综合服务水平。推进全民健身运动，办好市第五届运动会。全面完成经济普查工作，健全经济运行监测体系。做好宗教工作，促进民族团结。加强全民国防教育，重视驻军和民兵预备役建设，深入开展“双拥”活动，进一步密切军政、军民关系。继续做好侨务、老龄、对台、史志、档案等工作。

　　 （八）实施“三大战略”，扎实推进社会主义精神文明和民主、法制建设

　　实施可持续发展战略。继续抓好人口和计划生育工作，实现责任、措施、投入“三到位”，稳定低生育水平，优化人口结构，提高出生人口素质。全面落实环境保护目标责任制和一票否决制，严格实施《环境影响评价法》和新改扩建项目“三同时”制度。扎实推进“空心村”整治和土地保护工作，提高利用效率。积极推行清洁生产，建设资源节约型社会。认真实施天然林保护工程，抓好自然保护区、风景名胜区建设，改善生态环境。

　　实施科教兴市战略。加强产学研合作，组织实施科技攻关，加快科技成果的引进和推广，促进转化为现实生产力。坚持三教统筹，巩固“两基”成果，强化素质教育，提高教学质量。完善农村义务教育管理体制，落实“三保”政策，完成中小学D级危房改造6.5万平方米，积极推进现代远程教育。统筹规划整合教育资源，调整市区中小学布局。加快发展高中等职业技术教育，全面提高劳动者素质。

　　实施人才强市战略。加强人才队伍建设，创新工作机制，组织实施“112”工程，培养拔尖人才。充分发挥现有人才的作用，继续吸引优秀人才，拓展引进国外智力渠道，力求急需人才引得来、留得住、用得好。坚持“凡进必考”原则，充实公务员队伍，全面推进事业单位人事制度改革。科学开发和配置人才资源，促进人才合理流动。

　　加强社会主义精神文明建设。全面实施公民道德工程，深化“双创”和文明单位创建活动，加强精神文明基础设施建设，净化未成年人的成长环境。崇尚科学，反对迷信，倡导文明健康的生活方式。坚持“三贴近”和“三创新”，繁荣和发展文学艺术、广播电视和新闻出版事业。加强文化市场特别是互联网等现代传媒的管理，积极扶持群众性文化活动，搞好文物保护和利用，大力发展文化产业，满足人民群众日益增长的精神文化需求。

　　加强社会主义民主政治建设。各级政府及其工作人员要执行同级人大及其常委会的决议、决定，坚持重要工作报告制度，主动接受人大监督；积极支持人民政协履行政治协商、民主监督、参政议政职能，广泛听取社会各界的意见和建议。认真办理人大代表建议和政协委员提案，完善科学民主决策机制，提高决策透明度和公众参与度。切实加强与各民主党派、工商联和无党派人士的联系，充分发挥工会、共青团、妇联等人民团体的桥梁纽带作用。完善村民居民自治制度，扎实做好第五届村民委员会换届选举工作。

　　加强社会主义法制建设。坚持依法治市，加大普法力度，增强全民法制意识。加强社会治安综合治理，完善社会治安防控体系，争创综合治理先进市。依法严厉打击各种刑事犯罪和邪教组织的破坏活动，维护正常社会秩序。高度重视安全生产工作，把安全生产摆在与经济发展同等重要的位置，从制度、技术、投入和责任等方面采取措施，坚决遏制重特大事故的发生。完善正确处理人民内部矛盾的工作机制，认真做好群众来信来访工作，把各种矛盾和问题消化在基层，解决在萌芽状态。健全社会预警体系和突发公共事件应急机制，提高保障公共安全和处置突发事件的能力，努力维护社会大局稳定，全力打造“平安三门峡”。

三、努力建设服务型政府

　　各级政府和广大公务员必须认清在全面建设小康社会、跻身中原崛起“第一梯队”进程中所担负的历史重任，自觉适应新形势，不断加强自身建设，坚持做到“六个必须”，进一步提高行政能力和工作水平。

　　必须做到“三好一强”。把开展“三好一强”活动与保持党员先进性教育活动紧密结合起来，抓住学习“三个代表”重要思想这条主线，把握保持党员先进性这个主题，加强思想、作风、组织建设，增强各级政府机关公务员的责任感和使命感，激发积极性和创造力，为加快经济发展和构建和谐社会提供坚强的政治和组织保证。

　　必须转变政府职能。继续履行经济调节、市场监管职能，注重强化社会管理和公共服务职能，真正把政府职能转到为市场主体服务和创造良好环境上来。深化行政审批制度改革，健全行政服务网络。实行政企、政事分开，创新政府管理方式，抓好电子政务和信息资源开发，努力提高行政效能。全面推行政务公开，提升政府公信力。

　　必须提高执政能力。各级政府、各部门的领导干部要进一步增强驾驭市场经济和应对复杂局面的能力，注重学习和锻炼，自觉把党和国家的路线、方针、政策与工作实际结合起来，正确处理宏观调控与经济发展、经济发展与社会进步的关系，充分发挥主观能动性，创造性地开展工作，在调控中谋求经济社会协调发展。坚持科学执政、民主执政，建立与群众利益密切相关的重大事项社会公示、听证制度。深入研究改革发展稳定中的重大问题，科学制定“十一五”规划和2020年远景规划，为中长期发展打好基础。

　　必须坚持依法行政。各级政府、各部门及其工作人员要自觉遵守宪法和法律，依法行使权力、履行职责。重视政府法制工作，改进行政执法，严格实行行政执法责任制和执法过错责任追究制。进一步加强对行政权力的监督，规范行政行为，做到有权必有责，用权受监督，侵权要赔偿，违法要追究。

　　必须加强政风建设。牢固树立正确政绩观和科学发展观，加快建立和完善新的经济社会发展综合评价体系和干部政绩考核评价体系，大兴求真务实之风，继续下大力气精简会议、文件，最大限度地减少应酬和不必要的事务性活动。深入开展调查研究，了解社情民意，多为群众办实事、做好事。强化责任意识，狠抓工作落实，敢于坚持原则，敢于触及矛盾，敢于批评错误，反对好人主义，反对敷衍塞责，反对推诿扯皮。牢记“两个务必”，始终保持谦虚谨慎、不骄不躁、艰苦奋斗的优良作风。

　　必须抓好廉政建设。全面落实党风廉政建设责任制，严格执行廉政规定，做到廉洁从政，从严治政。坚持标本兼治、综合治理、惩防并举、注重预防的方针，建立健全惩治和预防腐败体系，加大经济责任审计力度，把反腐倡廉工作不断引向深入。加强行政监察工作，深化民主评议行风活动，切实纠正部门和行业不正之风，维护政府机关形象。

　　各位代表：

　　今年是贯彻落实科学发展观、保持经济社会良好发展态势的关键一年，也是全面实现“十五”目标、衔接“十一五”规划的重要一年。我们面临的任务艰巨而光荣，肩负的使命重大而神圣。让我们在以胡锦涛同志为总书记的党中央领导下，高举邓小平理论和“三个代表”重要思想伟大旗帜，坚定信心，同心同德，扎实工作，开拓进取，为实现全市经济社会全面协调可持续发展而努力奋斗！
